# The Flexible Action System: Click-Based Echolocation May Replace Certain Visual Functionality for Adaptive Walking

**DOI:** 10.1037/xhp0000697

**Published:** 2019-09-26

**Authors:** Lore Thaler, Xinyu Zhang, Michail Antoniou, Daniel C. Kish, Dorothy Cowie

**Affiliations:** 1 Department of Psychology, Durham University; 2 School of Information and Electronics, Beijing Institute of Technology; 3 Department of Electronic Electrical and Systems Engineering, School of Engineering, University of Birmingham; 4 World Access for the Blind, Placentia, California; 5 Department of Psychology, Durham University

**Keywords:** sonar, hearing, audition, multisensory, blindness

## Abstract

People use sensory, in particular visual, information to guide actions such as walking around obstacles, grasping or reaching. However, it is presently unclear how malleable the sensorimotor system is. The present study investigated this by measuring how click-based echolocation may be used to avoid obstacles while walking. We tested 7 blind echolocation experts, 14 sighted, and 10 blind echolocation beginners. For comparison, we also tested 10 sighted participants, who used vision. To maximize the relevance of our research for people with vision impairments, we also included a condition where the long cane was used and considered obstacles at different elevations. Motion capture and sound data were acquired simultaneously. We found that echolocation experts walked just as fast as sighted participants using vision, and faster than either sighted or blind echolocation beginners. Walking paths of echolocation experts indicated early and smooth adjustments, similar to those shown by sighted people using vision and different from later and more abrupt adjustments of beginners. Further, for all participants, the use of echolocation significantly decreased collision frequency with obstacles at head, but not ground level. Further analyses showed that participants who made clicks with higher spectral frequency content walked faster, and that for experts higher clicking rates were associated with faster walking. The results highlight that people can use novel sensory information (here, echolocation) to guide actions, demonstrating the action system’s ability to adapt to changes in sensory input. They also highlight that regular use of echolocation enhances sensory-motor coordination for walking in blind people.

## Public Significance Statement

Vision loss has negative consequences for people’s mobility. The current report demonstrates that echolocation might replace certain visual functionality for adaptive walking. Importantly, the report also highlights that echolocation and long cane are complementary mobility techniques. The findings have direct relevance for professionals involved in mobility instruction and for people who are blind.

There is a long line of research showing that people use sensory information, and in particular, vision, to make perceptual judgments (e.g., about object orientation) and to guide actions such as reaching for objects, or avoiding obstacles while walking. In this context, research suggests that visual perceptual judgments and action guidance are controlled by partially overlapping but nonetheless distinct neural systems (Milner & Goodale, 2006). While there has been considerable research on how the system may adapt and use novel nonvisual information for making perceptual judgments typically achieved using vision (e.g., judging shapes or sizes of distal objects), there has been comparably less work investigating this for visually guided actions (Bavelier & Neville, 2002; Burton, 2003; Maidenbaum, Abboud, & Amedi, 2014; Merabet & Pascual-Leone, 2010; Noppeney, 2007; Renier, De Volder, & Rauschecker, 2014; Röder & Rösler, 2004). Yet, since these functions may be served by different neural pathways it is not clear how results obtained with perceptual judgments may transfer and it is therefore important to examine how novel sensory information may be used to guide actions.

One challenge in investigating the capability of systems to integrate novel sensory information for certain behaviors and functions is to disentangle the effects of long-term sensory deprivation (e.g., vision loss) and experience in using another modality. For example, people who are blind will not only behave differently because of loss of vision, but also because they have had more experience in using nonvisual modalities such as touch or hearing to govern behavior. In this context, click-based echolocation is a suitable paradigm to investigate the influence of vision loss alongside the influence of experience in using a particular kind of sensory information.

Specifically, echolocation is the ability to infer spatial information based on sound reflections that arise from emissions being projected into the environment (Griffin, 1944). Echolocation is well known from certain species of bat and marine mammals, who typically echolocate using emissions in the ultrasonic range. Humans can also echolocate, using emissions in the audible spectrum, such as mouth clicks (for reviews, see Kolarik, Cirstea, Pardhan, & Moore, 2014; Stoffregen & Pittenger, 1995; Thaler & Goodale, 2016). At present, people rarely use click-based echolocation spontaneously. Yet, research has shown that it can be learned by both blind and sighted people (Ekkel, van Lier, & Steenbergen, 2017; Kolarik, Scarfe, Moore, & Pardhan, 2016, 2017; Schörnich, Nagy, & Wiegrebe, 2012; Teng & Whitney, 2011; Thaler, Wilson, & Gee, 2014; Tonelli, Brayda, & Gori, 2016; Tonelli, Campus, & Brayda, 2018; Worchel & Mauney, 1951). Importantly, people who do not use click-based echolocation on a regular basis will be new to this skill regardless of vision loss. Of key importance here is that there are currently a small number of people worldwide who are blind and use click-based echolocation all the time, and as a consequence have considerable experience with it. As such, a comparison of these blind echolocation experts with participants new to echolocation can show to what degree changes in behavior are due to sensory deprivation (i.e., blindness) or experience with using a particular type of sensory information (i.e., click-based echolocation). This gives us the opportunity to investigate to what degree behaviors that are typically achieved using vision could also be achieved using other sensory information.

It has been shown that people who are blind, as well as sighted people who have been blindfolded, can successfully guide their walking using sensory-substitution devices such as the sonic torch (Kolarik et al., 2017), or mouth clicks (Kolarik et al., 2016, 2017; Tonelli et al., 2018). Using a 3D motion capture system Kolarik et al. (2016, 2017) also found that while echolocation aided successful avoidance of obstacles, participants new to echolocation walked slower than sighted participants using vision (Kolarik et al., 2016, 2017). This exemplifies that while people were able to generally control their walking using click-based echolocation, this was associated with impaired walking speed. Kolarik et al. (2017) also tested a single blind person who was reported to use echolocation in daily life, but it was not stated when they had started using it. This participant performed not significantly different from blind people new to echolocation, and showed reduced walking speed compared to people who were sighted. This result is somewhat surprising and seemingly at odds with reports in the public media showing blind expert echolocators walking with sighted people with equal speed, or even running or riding bicycles. Furthermore, previous research using perceptual judgments has shown that experience with click-based echolocation is associated with improved performance when perceptually judging the shape, size, or distance of objects on the basis of echoes from mouth clicks (Fiehler, Schütz, Meller, & Thaler, 2015; Milne, Anello, Goodale, & Thaler, 2015; Milne, Goodale, & Thaler, 2014). Indeed, blind echo experts perform similarly to sighted people using vision when judging the weight of objects (Buckingham, Milne, Byrne, & Goodale, 2015), the physical size of objects (Milne et al., 2014) or the relative location of sounds with respect to one another (Vercillo, Milne, Gori, & Goodale, 2015). As such, there is the possibility that Kolarik et al.’s (2017) results might not be representative of a larger sample of echolocation experts. In sum, a key remaining question is if experience with echolocation would improve participants’ ability to use this “novel” sensory cue for action guidance. We here address this question using an adaptive walking task and click-based echolocation with samples of blind and sighted participants including echolocation experts.

To maximize the relevance of our research for people with vision impairments we decided to include the long cane as an additional variable, and we considered obstacles at different elevations. This will enable us to determine the usefulness of echolocation in a natural setting (i.e., with long cane and without) and across conditions relevant to people with vision loss (i.e., obstacles on the ground vs. obstacles at head height). To investigate how movement kinematics and click acoustics are related, we acquired 3D-movement and sound data simultaneously.

To investigate the effects of blindness and experience in echolocation, we had three different participant groups: Blind echolocation experts (*n* = 7), blind echolocation beginners (*n* = 10), and sighted echolocation beginners (*n* = 14). During an obstacle avoidance task, the obstacle could be either at head height or on the ground. People completed trials without any vision (i.e., blind or blindfolded), but using echolocation, long cane, or both. There were also trials where sighted people used vision (*n* = 10). In this way, we could compare the functional equivalence between echolocation and vision.

Following previous research on adaptive walking with echolocation in blindfolded sighted people (Kolarik et al., 2016, 2017) we used movement speed and number of collisions as kinematic measures. We further characterized walking performance using movement deceleration and average movement paths. We characterized acoustic performance using click rate, click duration, intensity, and peak spectral frequency.

## Method

All procedures were approved by the Durham University Department of Psychology Ethics Committee (Ref. 14/13) and adhered to the Declaration of Helsinki and the British Psychological Society code of practice. All participants gave written, informed consent prior to participating in the study. Blind participants received accessible versions of all documents. The locations for signing were indicated via visual and tactile markers.

### Participants

A total of 41 people participated in the study: 24 sighted participants new to echolocation (14 in echolocation and cane conditions: 6 male; *M*_age_ = 22.4 years, *SD* = 6.2 years, range 18–41 years; 10 in vision conditions: 3 male; *M*_age_ = 26.3, *SD*: 6.8, min 19, max 38), 10 blind participants (Table 1), and seven blind echolocation experts (Table 2). Sample sizes were driven by availability of participants (blind echo experts) and previous work in this area (blind echo beginners, sighted participants). Independent samples *t* tests showed that while there was no significant age difference between sighted vision and sighted blindfolded participants, *t*(22) = 1.480; *p* = .153, and between blind participants new to echolocation and blind participants with experience in echolocation, *t*(15) = 1.974; *p* = .067; blind participants overall were significantly older than sighted participants, *t*(39) = 6.080; *p* < .001. All participants had normal hearing and no history of neurological disorder. All blind participants were independent travelers. All participants (except sighted in vision conditions) were tested with blindfold to ensure consistent testing conditions.[Table-anchor tbl1][Table-anchor tbl2]

### Apparatus and Setup

The study was carried out at Durham University Psychology Department. The testing room measured 5.8 m (W) × 9 m (L) × 3 m (H), with carpeted flooring, and walls lined with black fleece curtains. The wall toward which participants were walking was additionally covered with 5-in.-deep acoustic foam pads. The noise level in the room was ∼40 dBA. The long cane was an Ambutech (Ambutech, Winnipeg, Canada) telescopic graphite long cane with ceramic tip. The length was adjusted to the height of each participant, such that it measured from the floor to the height of their sternoclavicular joint. The reflective coating of the cane was covered with tape.

The lab was equipped with a 16-camera Bonita Vicon motion capture system running at 240 Hz, and Vicon Nexus 1.8.5 software (Vicon, Oxford, UK). Movements were tracked via 12-mm infrared-reflective markers. Markers were placed on the left and right lateral malleoli (ankles), second metatarsal heads (toes), heels, and acromion processes (shoulders). An “extra” marker was placed anterior to the ankle marker on the arch of the right foot. Six markers were mounted on an elastic tape “helmet” worn on the head (2 markers placed on the right side of the head, 2 on the left side, 1 at the back, and 1 on the top of the head). Markers were also placed on the long cane, with one just above the cane tip, one 20 cm distant from the cane tip, and one below the handle. Two 80 cm × 80 cm polystyrene obstacles were used in the study. For both obstacles, the plane facing the participant was painted with primer; the plane facing away was covered with black felt. The top obstacle was suspended from the ceiling with strings. For each participant the height of the top obstacle was adjusted so that the center of the object matched the height of their mouth. The bottom obstacle was placed on the ground and remained upright by itself. Markers were attached to each of the front corners of each obstacle. Obstacles were placed at three different distances directly ahead (2.1 m, 3.05 m, and 4 m) of the participant’s starting position. The starting position was positioned 2.75 m into the room lengthwise, and 2.9 m into the room widthwise (i.e., in the middle of the room). From their starting position the participant walked the length of the room toward the opposite wall. Thus, the distance between the final obstacle and the opposite wall of the room was 2.25 m. These distances were chosen to enable participants (in trials using a cane) to make unobstructed use of the cane from beginning to end of any trial. Participants’ clicks were recorded with Tascam DR-100 MK2 (24-bit 96 kHz; Tascam, TEAC Corporation, Japan) which was kept in a hip pack attached to the participant and DPA SMK-SC4060 (DPA microphones, Alleroed, Denmark) omnidirectional binaural microphones worn next to each ear. A blindfold was used for each participant, and participants were required to wear earplugs (3M EAR UF-01–014, 3M, Maplewood, Minnesota, US) during the interval between trials to avoid gaining auditory information about obstacle placement.

### Design

The between-subjects variable was group, that is, blind experts, blind beginners, sighted blindfolded beginners, and sighted (using vision). For participants in echolocation/cane conditions, the within-subjects variables were obstacle location (head height vs. ground level) and method (echolocation, cane, echolocation and cane). For sighted participants in vision conditions, the within-subjects variables were obstacle location (head height vs. ground level) and method (vision, vision and cane). To prevent the task from being predictable, obstacles in all conditions and groups could be presented at various distances (2.1 m, 3.05 m, and 4 m) and also be absent (see also the following Task and Procedure sections).

### Task and Procedure—Echolocation and Long Cane Conditions

All participants (blind and sighted) wore a blindfold at all times during the experiment. Before each individual trial, participants were instructed to block their ears using earplugs, and to hum, in order to block any remaining sounds possibly arising from obstacle placement. The obstacle was placed by the experimenter. In trials where no obstacle was present, or where the obstacle remained in the same position, the experimenter walked and pretended to place an obstacle. Once this had been done, the experimenter signaled the start of a trial to the participant. Participants then stopped humming, removed the earplugs, and commenced the trial.

In each trial the task was to walk from their starting position toward the opposite end of the room. Participants were told that, should they sense that there was an obstacle in front of them, they were to walk around it without touching it with any part of their body, but otherwise they were to continue walking straight ahead. Participants were told that they should walk the way they would usually walk, that is, with a speed and movement pattern that felt natural to them. They were told that they had as much time as they wanted. They were told to keep walking until the experimenter said “stop.” The experimenter stopped the trial either when the participant had avoided/collided and then walked past the obstacle or (in trials when no obstacle had been present) when the participant had walked past the distance of the furthest obstacle. Upon completion of the trial the experimenter guided the participant back to their starting position (marked with Velcro strips on the floor).

Each participant in the beginner group completed a training session (lasting 1 hr). During the training session, participants were instructed on how to make mouth clicks, and how to use echolocation to detect that something is in front of them, and to move around an obstacle. A detailed description of the training procedure is given in the online supplemental materials. Sighted participants were also introduced to using the long cane (“sweeping” motion going from left to right and vice versa at about the width of a person’s shoulders in front of them on ground level; a constant contact technique commonly taught by mobility instructors in the United Kingdom). Then, we explained the basic structure of the experimental trials. Once they had gained a working knowledge of the process, they completed at least 12 practice trials that mirrored those used in the experimental conditions. At the end of the training session participants were invited for the two further experimental sessions to take place on separate days. Participants in the blind expert group were also introduced to the task as beginner groups. However, due to their expertise, a separate training session was not required.

The actual experiment then consisted of two separate sessions each lasting 2 hr. Each session contained 51 trials total. Broken down by condition, it contained two trials per combination of distance (2.1 m, 3.05 m, and 4 m), obstacle height (ground level/head level), and method (echo/cane/both; 2 × 3 × 2 × 3 = 36 trials), and an additional five trials without any obstacle for each method (3 × 5 = 15). Conditions were presented in pseudorandomized order in each session. The trials without obstacles were included to avoid participants anticipating an obstacle on every trial, but were not analyzed further. Both sessions followed identical structures, excluding the trial order.

### Task and Procedure—Vision Conditions

The overall procedure was similar to those in echolocation and long cane conditions. In between trials, participants wore a blindfold and ear plugs, and hummed. The main difference was that participants removed both blindfold and earplugs before commencing a trial. In this set of trials, participants only encountered two conditions: vision only, and vision and long cane. Thus, there was no practice for echolocation, but participants were introduced to using the long cane just like participants in the echolocation and long cane conditions. The experiment consisted of one session lasting 1.5 hr. The session contained 48 trials total. Broken down by condition, it contained four trials per combination of distance (2.1 m, 3.05 m, and 4 m), location (top/bottom), and method (vision/vision and cane; 4 × 3 × 2 × 2 = 48 trials). Conditions were presented in pseudorandomized order.

### Analysis of Kinematic Data

Each individual trial was quality checked by the experimenter, to ensure all markers were labeled correctly by the motion capture system. Following Kolarik et al. (2016, 2017), a trial was recorded as containing a collision if any part of the participant’s body touched the obstacle during the trial. This was determined by audio-visual inspection of each trial. Data were exported in .csv format for further analysis in Matlab (Version R2012a; MathWorks, Natick, MA). Gaps in labeling were dealt with in Matlab either by completing gaps based on positions of other markers (where missing markers were part of a rigid body configuration) or by cubic-spline interpolation. Following Kolarik et al. (2016, 2017), participant movement speed was measured for the final meter in the forward dimension before colliding with the obstacle (for trials in which a collision occurred), or before crossing the front aspect of the obstacle with their head (for trials in which no collision occurred). Deceleration was computed as the difference in movement speed between the ultimate and the penultimate meter before the obstacle. We chose to focus all analyses on the motion of the head because initial analyses had shown that the head was the best available indicator of whole-body speed and position. Each of these kinematic measures was averaged across obstacle distances and trials for each condition and participant. We averaged across distances, because results did not differ across distances. To further characterize behavior we also calculated the average walking paths that participants took during successful avoidance of obstacles. Average walking paths were calculated based on low pass (30 Hz) filtered individual traces. Each filtered walking path was split into 400 segments of equal length from start of the movement until the obstacle was crossed. Then, the average path was calculated by averaging across coordinates corresponding to each path segment. While participants avoided the obstacle both to the left- and the right-hand sides, we mirrored walking paths to the left-hand side along the horizontal axis to facilitate calculation of averages (i.e., after mirroring of left side paths all paths went to the right-hand side).

### Analysis of Acoustic Data

Acoustic data were not available for one blind echo beginner due to technical difficulties. For trials where participants had made clicks (i.e., echolocation, and echolocation and long cane) we extracted the peak frequency for each click, click duration, clicking rate, and root mean square intensity. To calculate clicking rate, the sound was analyzed from the time when the first click occurred, until either colliding with the obstacle (for trials in which a collision occurred), or before crossing the front aspect of the obstacle (for trials in which no collision occurred). Analyses were done in Matlab (Version R2012a; MathWorks, Natick, MA). Clicks were detected using visual inspection, and start and end were defined as those points in time where the signal envelope reached 1% of the maximum magnitude.

### Statistical Analyses

Data were analyzed using SPSSv22. To compare data across the four groups (sighted vision, blind experts, blind beginners, sighted beginners) we averaged across methods and locations. These data were subsequently analyzed with factorial analysis of variance (ANOVA). To investigate the effects of location and method we used repeated or mixed model ANOVA as appropriate. Where the sphericity assumption could not be upheld, Greenhouse-Geisser (GG) correction was applied. Any post hoc tests used Bonferroni correction. Average walking paths were compared using 95% confidence intervals around the average trace.

## Results

### Kinematic Data

#### Collisions

**Figure 1 fig1:**
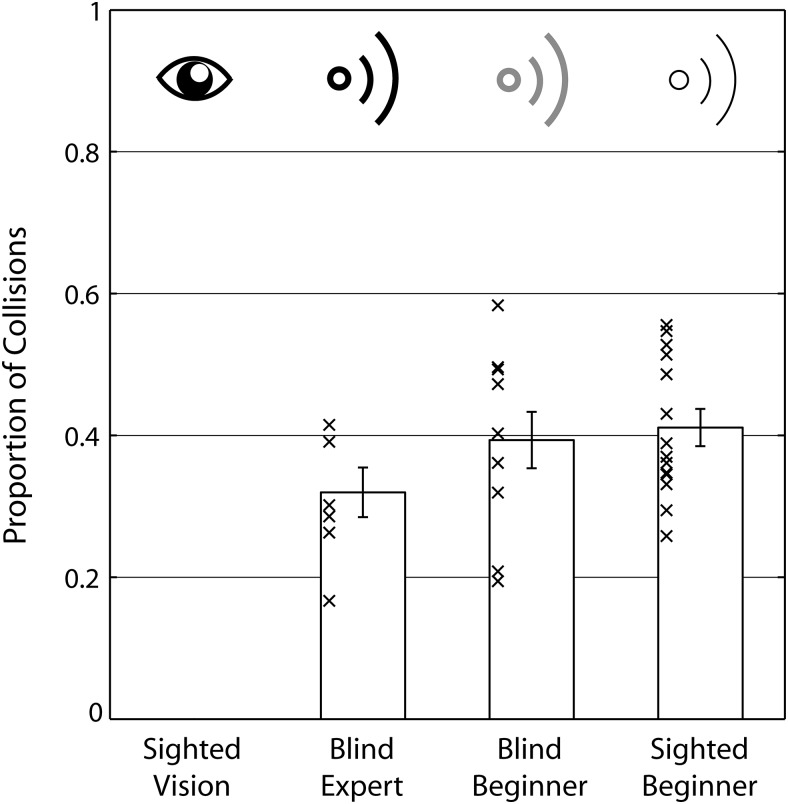
Collisions averaged across conditions for the four different groups. Sighted participants using vision had no collisions. Bars are group means, errors bars are *SEM* across participants, and crosses are individual participant’s data points. Sighted people using vision had no collisions.

Figure 1 shows how often each participant group collided with the obstacle. Sighted participants using vision had no collisions. An ANOVA with the between-subjects variable group was significant, *F*(3, 37) = 43.877; *p* < .001; η_*p*_^2^ = .781. Post hoc pairwise comparisons showed that sighted people using vision had fewer collisions than any of the other participant groups (for all groups *p* < .001), while differences across blind experts, blind, and sighted beginners were all nonsignificant (experts vs. blind beginners: *p* = .712; experts vs. sighted beginners: *p* = .265; blind beginners vs. sighted beginners: *p* < .999).[Fig-anchor fig1]

**Figure 2 fig2:**
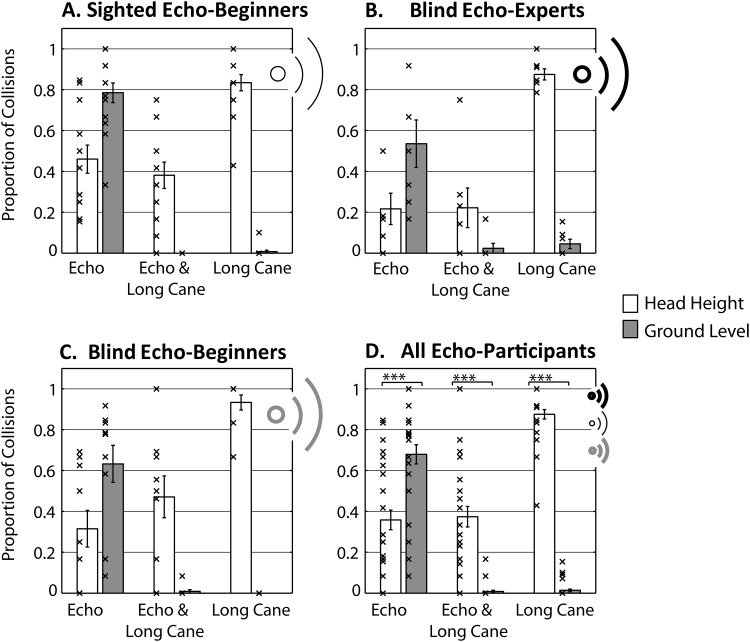
Collision data across conditions. Bars are group means, errors bars are *SEM* across participants, and crosses are individual participant’s data points. Sighted people using vision had no collisions. *** *p* < .001 (Bonferroni corrected).

Figure 2 shows collision data for the different conditions and groups. An initial mixed ANOVA was applied to data from those participants who had worked in the absence of vision (blind experts: Figure 2B; blind beginners: Figure 2C; sighted beginners: Figure 2A) with method and location as repeated variables and group as between-subject variable revealed that the effect of location was significant, *F*(1, 28) = 98.232; *p* < .001; η_*p*_^2^ = .778, and the effect of method was significant, *F*(2, 56) = 48.327; *p* < .001; η_*p*_^2^ = .633. People had generally fewer collisions for obstacles at ground level (*M* = .23, *SD* = .09) as compared to head height (*M* = .52, *SD*= .17), and they had fewer collisions when using echolocation and cane combined (*M* = .18, *SD*= .14) as compared to echolocation only (*M* = .49, *SD*= .23; *p* < .001) or cane only (*M* = .45, *SD*= .07; *p* < .001), but there was no difference between cane and echolocation only (*p* = .886). Importantly, main effects were moderated by a significant interaction effect between location and method, *F*(2, 56) = 219.298; *p* < .001; η_*p*_^2^ = .887 and between group and method, *F*(4, 56) = 4.111; *p* = .005; η_*p*_^2^ = .227. No other effects were significant.[Fig-anchor fig2]

To follow up the interaction effect of location and method, we removed group as a variable and computed two repeated measures ANOVAs to compare numbers of collisions across methods separately for top and bottom obstacles. Both analyses revealed significant effects of method on collisions (top: *F*(2, 60) = 69.678, *p* < .001, η_*p*_^2^ = .699; bottom: *F*_GG_(1.029, 30.863) = 194.150, *p* < .001, η_*p*_^2^ = .866). Post hoc comparisons showed that for the top obstacle, numbers of collisions were highest when people used the cane only (*M* = .88; *SD*= .12) as compared to when they used echolocation only (*M* = .36; *SD*= .27; *p* < .001), or echolocation and cane (*M* = .37; *SD*= .28; *p* < .001), but that there was no significant difference between echolocation and cane and echolocation (*p* = .999). Overall, therefore, the use of echolocation enabled participants to reduce number of collisions for the head level obstacle by ∼52%. For the bottom obstacle, we found that numbers of collisions were highest when people used echolocation only (*M* = .68; *SD* = .26), as compared to when they used the cane only (*M* = .01; *SD* = .03; *p* <.001), or the cane together with echolocation (*M* = .01; *SD* = .03; *p* <.001). There was no significant difference between cane only and cane and echolocation (= .999).

To follow up the interaction effect between group and method, we subsequently computed three one-way ANOVAs comparing number of collisions across groups, for each method separately. We found that there was a significant effect of group on number of collisions when people used echolocation, *F*(2, 28) = 3.508; *p* = .044; η_*p*_^2^ = .200, but not when using either cane, *F*(2, 28) = 1.919; *p* = .166; η_*p*_^2^ = .121, or cane and echolocation, *F*(2, 28) = 1.530; *p* = .234; η_*p*_^2^ = .099. Yet, even though echolocation experts had overall the lowest number of collisions when using echolocation (*M* = .38, *SD* = .20) as compared to sighted (*M* = .62; *SD* = .19) and blind beginners (*M* = .47; *SD* = .24), none of the post hoc pairwise comparisons (Bonferroni) were significant.

#### Movement speed

**Figure 3 fig3:**
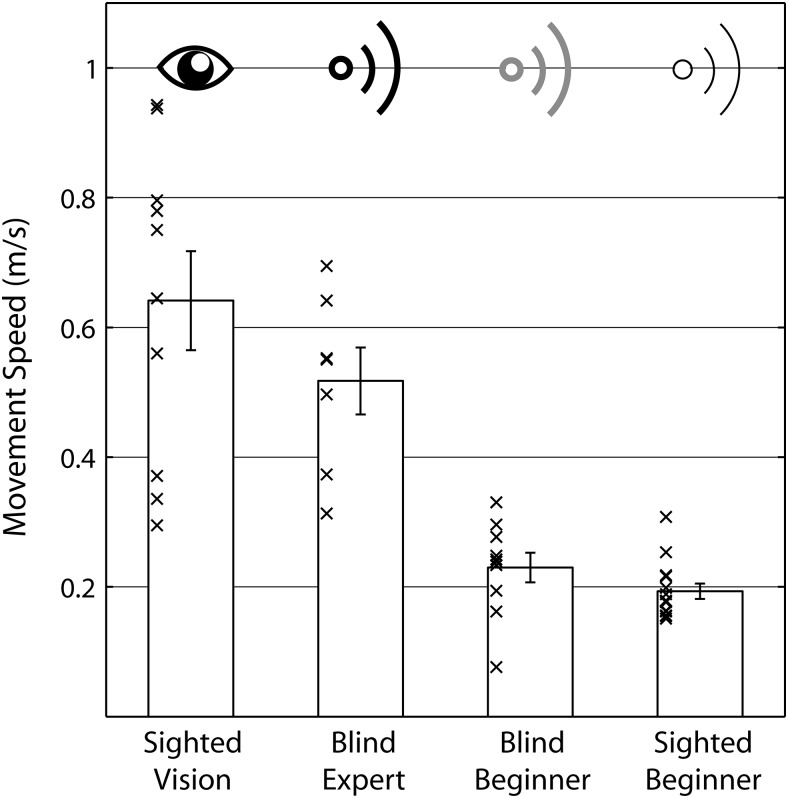
Movement speed averaged across conditions for the four different groups. Bars are group means, errors bars are *SEM* across participants, and crosses are individual participant’s data points.

Movement speed for the four different groups averaged across conditions is shown in Figure 3.^1^ An ANOVA with the between-subjects variable group was significant, *F*(3, 37) = 26.407; *p* < .001; η_*p*_^2^ = 682. Post hoc pairwise comparisons (Bonferroni) across groups showed that speed did not differ significantly between sighted participants using vision and blind echo experts (*p* = .469) or between sighted and blind echo beginners (*p* = .999). But speed was significantly higher for blind echo experts than for blind echo beginners (*p* = .001) or sighted echo beginners (*p* < .001). Average speed was also significantly higher for sighted people using vision than for sighted echo beginners (*p* < .001) or blind echo beginners (*p* < .001).[Fig-anchor fig3]

**Figure 4 fig4:**
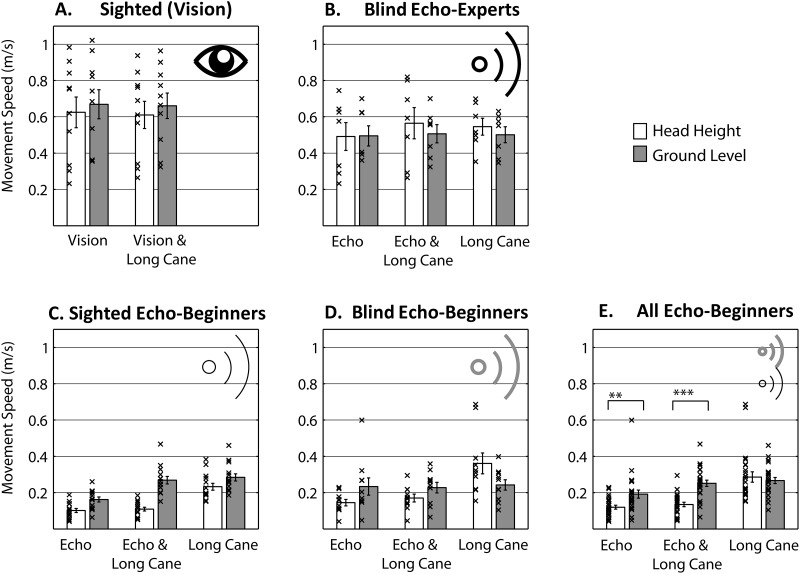
Movement speed across conditions. Bars are group means, errors bars are *SEM* across participants, and crosses are individual participant’s data points. ** *p* < .01. *** *p* < .001 (Bonferroni corrected).

Figure 4 shows movement speed data broken down by condition and group. An initial mixed ANOVA was applied to all data from the echolocation groups. Method (echo, long cane, both) and obstacle height (ground, head) were within-subjects variables, and group (blind echo experts, blind echo beginners, sighted echo beginners) was a between-subjects variable. This revealed a significant interaction between group, method, and obstacle height (*F*_GG_(2.609, 36.520) = 4.013; *p* = .018; η_*p*_^2^ = .223). To follow up this interaction we subsequently investigated across groups. Performance of blind echo beginners (Figure 4C) and sighted echo beginners (Figure 4D) did not differ significantly across any conditions. We therefore combined data from these two beginner groups to increase statistical power (Figure 4E). We subsequently analyzed data for blind echo experts and all beginners separately using repeated measures ANOVA with method and location as factors. The same analysis was also applied to data from sighted participants using vision.[Fig-anchor fig4]

An ANOVA analysis did not reveal any significant effects for sighted people using vision (Figure 4A). Thus, movement speed of sighted people using vision was the same across obstacle locations and method used. The same result also applied for blind echo experts (Figure 4B). In contrast, for echolocation beginners the main effect of location was significant, *F*(1, 23) = 13.348; *p* < .001; η_*p*_^2^ = .367, the main effect of method was significant, *F*(2, 46) = 45.173; *p* < .001; η_*p*_^2^ = .663 and the interaction between location and method was significant (*F*_GG_(1.211, 27.863) = 8.119; *p* = .001; η_*p*_^2^ = .261). As is evident in Figure 4E, people moved more slowly when using echolocation or echolocation and cane, but this was more pronounced for the obstacle at head height. In sum, people who use echolocation on a regular basis walk at the same speed as people using vision, and faster than blind or sighted people new to echolocation. Therefore, the results suggest that regular use of echolocation may support sensorimotor coordination for walking in people who are blind. Or on a more general level, the results show that experience significantly improves the ability to guide actions using novel sensory information.

#### Deceleration

**Figure 5 fig5:**
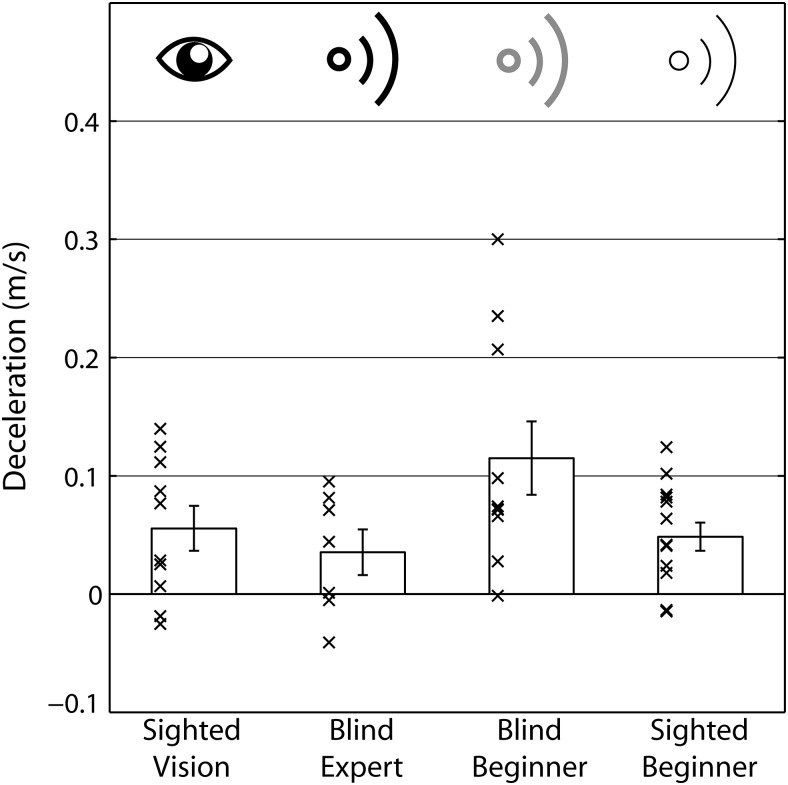
Deceleration data averaged across conditions for the four different groups. Larger values indicate more slowing down during approach of the obstacle. Bars are group means, errors bars are *SEM* across participants, and crosses are individual participant’s data points.

Figure 5 (see also Footnote 1) shows, by group, participants’ deceleration in the approach to the obstacle: that is, the difference in approach speed between ultimate and the penultimate meter before the obstacle. Positive values mean that participants slowed down. An ANOVA revealed a significant effect of the between subject variable group, *F*(3, 37) = 2.972; *p* = .044; η_*p*_^2^ = .194. Yet, none of the post hoc pairwise comparisons (Bonferroni) were significant. Overall, the pattern of results suggests that blind echo beginners have a tendency to slow down more than any of the other participant groups.[Fig-anchor fig5]

**Figure 6 fig6:**
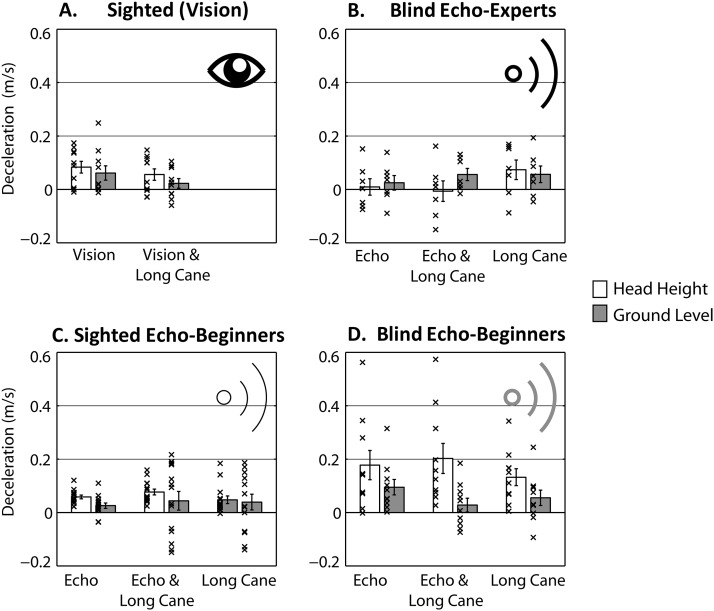
Deceleration data across conditions. Larger values indicate more slowing down during approach of the obstacle. Bars are group means, errors bars are *SEM* across participants, and crosses are individual participant’s data points.

Figure 6 shows participants’ deceleration broken down by condition and group. An initial mixed ANOVA applied to data from those participants who had worked in the absence of vision (blind experts: Figure 6B; blind beginners: Figure 6C; sighted beginners: Figure 6D) with method and location as repeated variables and group as between subject variable revealed a significant main effect of location, *F*(1, 28) = 6.944; *p* = .014; η_*p*_^2^ = .199, such that people had decelerated more for obstacles at head height (*M* = .088; *SD* = .10) as compared to obstacles at ground level (*M* = .046; *SD* = .07). Importantly, this was moderated by a significant interaction between group and location, *F*(2, 28) = 6.466; *p* = .005; η_*p*_^2^ = .316. None of the other effects were significant. To follow up this significant interaction we collapsed across method and subsequently investigated the effect of group (blind experts, sighted beginners, and blind beginners) separately for each obstacle location, by running a one-way ANOVA with the between-subject variable group separately for each obstacle location. We found that there were no differences in deceleration across groups for the obstacle at ground level, *F*(2, 28) = .324; *p* = .726; η_*p*_^2^ = .023. In contrast, deceleration differed across groups for the obstacle at head height, *F*(2, 28) = 7.156; *p* = .003; η_*p*_^2^ = .338. Post hoc tests (Bonferroni) showed that, for head-height obstacles, blind beginners slowed down significantly more (*M* = .17, *SD* = .13) than blind experts (*M* = .025, *SD* = .074; *p* = .006) and sighted beginners (*M* = .061, *M* = .036; *p* = .014), while the difference between sighted beginners and blind experts was not significant (*p* < .999).[Fig-anchor fig6]

A repeated measures analysis with method and location was also applied to data from sighted participants using vision (Figure 6A). This revealed a significant effect of location, *F*(1, 9) = 6.241; *p* = .034; η_*p*_^2^ = .409, such that people had slowed down more for obstacles at head height (*M* = .069, *SD* = .07) as compared to obstacles at ground level (*M* = .042; *SD* = .06). No other effects were significant.

In sum, all participants slowed down more for the obstacle at head height, but blind echo beginners slowed down more in this condition than the other groups.

#### Walking paths during obstacle avoidance with echolocation

**Figure 7 fig7:**
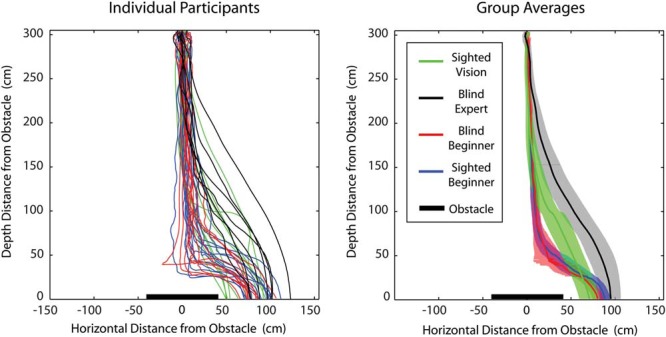
Walking paths averaged across trials were people used echolocation to successfully avoid obstacles. Participants avoided obstacles both to the left- and the right-hand sides. To facilitate calculation of averages walking paths from the left-hand side were mirrored to the right-hand side. Individual participant’s traces are shown in the top left panel. The top right panel shows group averages with shaded areas signifying 95% confidence intervals around the mean.

To investigate how people moved when they used echolocation to avoid obstacles we calculated the average walking paths for conditions where people used either clicks only or clicks and cane to avoid the obstacle at head level. For the other conditions there were either too few successful (i.e., noncollision) trials for reliable estimates of walking paths, or people relied on the cane. Figure 7 (top left) shows walking paths for individual participants, color coded by group, while the top right panel shows average paths by group, and in shaded areas 95% confidence intervals around the average. It is evident from the figures that there are differences across groups in terms of their walking paths. Specifically, blind echo experts compared to both sighted and blind echolocation beginners adjust their walking paths earlier to avoid the obstacle. The same pattern can be observed for sighted people using vision as compared to echolocation beginners; that is, sighted people using vision initiate adjustments earlier. Interestingly, when directly comparing blind echo experts and sighted people using vision, while both groups appear to initiate the adjustment early, the adjustment made by the blind echo experts is wider, that is, they leave a larger gap toward the obstacle, as compared to sighted people using vision. This can be understood as a possible safety margin, possibly because echoes might provide less precise localization of the obstacle as compared to vision. In sum, walking paths of blind echo experts differ from those of people who are new to echolocation, and instead more closely resemble walking paths by sighted people using vision. In conditions where no obstacle had been present participants had walked the length of the room in a straight line. There was no difference in movement paths on these trials across participant groups.[Fig-anchor fig7]

### Acoustic Data and Relationship to Kinematic Data

Table 3 shows summary statistics of sounds that people made in the different groups. These values are consistent with previous data on clicks used for echolocation (De Vos & Hornikx, 2017; Schörnich et al., 2012; Thaler & Castillo-Serrano, 2016; Thaler et al., 2017). An ANOVA with group as factor showed that clicking rate and loudness did not differ across groups, but there was a significant effect of group on peak spectral frequency, *F*(2, 27) = 7.167; *p* = .002; η_*p*_^2^ = .361 and duration, *F*(2, 27) = 6.761; *p* = .004; η_*p*_^2^ = .334. Post hoc pairwise comparison (Bonferroni) showed that peak spectral frequency was higher for echo experts as compared to sighted echo beginners (*p* = .002), and that duration was shorter for blind experts as compared to sighted beginners (*p* = .011) and also shorter for blind beginners as compared to sighted beginners (*p* = .024). No other comparisons were significant.[Table-anchor tbl3]

**Figure 8 fig8:**
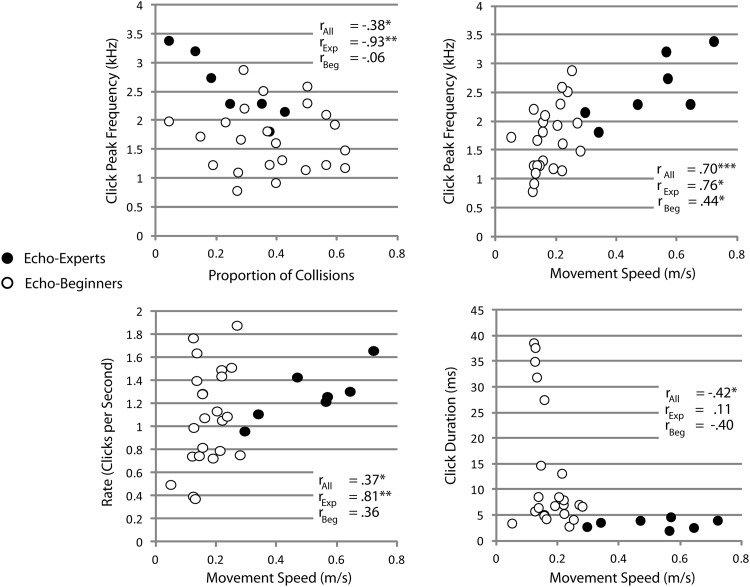
Scatter plots between acoustic click data and kinematics where significant pairwise correlations were found. Black symbols are data from echo experts. * *p* < .05. ** *p* < .01. *** *p* < .001.

To investigate potential relationships between the sounds that people made and their movements we correlated acoustic parameters of clicks with movement speed, deceleration, and collisions averaged across echolocation conditions (i.e., for this analysis we left out cane-only trials because people had not made any clicks for those). Scatterplots and significant correlations are shown in Figure 8, with data from echo experts highlighted in black. With respect to collisions, it appears that people who had fewer collisions also had a tendency to make brighter clicks (i.e., clicks with higher peak frequency), but considering experts and beginners separately, it seems that this relationship only holds for experts. With respect to movement speed the results suggest that considering all participants together, people who walked faster had a tendency to make brighter and briefer clicks at a higher rate. Yet, considering experts and beginners separately, for experts it can be said that those who walked faster also made brighter clicks at higher rates, while for beginners it can only be said that those who walked faster also made brighter clicks. In sum, brighter clicks were generally associated with better performance. This is consistent with previous reports in the literature (Norman & Thaler, 2018; Thaler & Castillo-Serrano, 2016; Thaler et al., 2017), but in contrast to those previous reports that focused on a perceptual judgments the current finding is the first to relate click acoustics to movement during walking.[Fig-anchor fig8]

### Acoustic Data and Expertise and Relationship to Kinematic Data

To investigate if expertise or blindness contributes to performance above and beyond acoustics of clicks, we calculated hierarchical multiple linear regression analyses, one each to predict each of the kinematic measures that correlated with acoustics (collisions; movement speed). For each analysis, the acoustic predictors shown in Figure 8 (peak frequency, rate, and duration) were used to predict kinematic performance. In addition, we included two dummy variables. One coded echolocation expertise (experts vs. nonexperts) and one variable coded blindness (sighted vs. blind). For movement speed we found that adding these two dummy variables made a significant contribution to the model (*R*^2^ change = .337; *F*(2, 24) = 22.705; *p* < .001) resulting in an overall *R*^2^ of .822, *F*(5, 24) = 22.166; *p* < .001. In the final model peak frequency (standardized beta = .311; *t*(24) = 2.370; *p* = .026) and expertise (standardized beta = .722; *t*(24) = 6.117; *p* < .001) contributed significantly, while none of the other variables (including the variable coding for blindness) were significant. The same type of analysis did not reveal any significant contributions of expertise or blindness on collisions.

## Discussion

### The Flexible Action System—Kinematics, Blindness, and Expertise

All participants, blind and sighted, were able to do the tasks in our experiment and were able to use click-based echolocation successfully to improve detection and avoidance of obstacles. While sighted people using vision made no collisions at all, there were still collisions for blind and blindfolded participants using echolocation, but the use of this technique enabled them to reduce collision rates for the obstacle at head height on average by ∼52% compared to when they were not using echolocation (i.e., long cane conditions). This is consistent with previous research, showing that echolocation provides sensory benefits in low vision conditions in adaptive walking tasks even for blind and sighted people who are new to using this type of sensory information (Kolarik et al., 2016, 2017). Yet, our study is the first to systematically examine whether expertise can strengthen the coupling between echolocation and walking, improving behavior in an adaptive walking task. Importantly, we examine the effects of expertise above and beyond the effects of blindness. We achieve this by including data from a group of blind experienced echolocators, as well as blind and sighted echo beginners. Analysis of movement speed showed that blind echo experts walked more quickly than blind and sighted blindfolded beginners, and, in fact, just as quickly as sighted participants. Similarly, walking paths of echolocation experts indicated early and smooth adjustments for avoidance of obstacles, similar to those by sighted people using vision and different from later and more abrupt adjustments of beginners.

These results confirm not only that the brain can adapt to use this new type of sensory information to guide actions such as adaptive walking behavior in beginners (i.e., successfully avoid obstacles), but importantly they suggest that with experience the brain might even replace visual functionality with other sensory information to govern certain parameters of actions (i.e., walking speed, walking paths).

Importantly, our study further showed that differences in walking speed are not limited to trials where people used echolocation, but also applied in trials that used the cane only. The fact that echolocators’ deceleration does not differ across conditions shows that even though they slow down in approach to the obstacle just like the other participants, they still continue to move at a higher average speed. The finding that experts move at higher speeds (indeed, as fast as sighted people using vision) might be because of benefits of long-term echolocation on sensory-motor coordination even when they are not using clicks at that very moment. For example, they may have a better impression of the space they are moving in stored in their memory from those trials where they did use the clicks. This might then translate into benefits even in trials where they are not using clicks. In our study trials with and without clicking were randomly mixed throughout the experiment. Future research could investigate the role played by spatial memory in more detail, for example, by studying kinematics of movements in echolocation experts and echolocation beginners under longer periods of nonclicking.

In our study we did not have a group of sighted echolocation experts. Yet, the pattern of results we obtained does highlight the important role played by echolocation expertise on performance in our study. Specifically, beginners who are blind performed like beginners who are sighted, but both beginner groups differed from the experts who are blind. Since the only difference between the two blind groups was expertise, while the only difference between the two beginner groups is long-term adaptation to vision loss, this pattern of results is consistent with the idea that expertise rather than long-term adaptation to vision loss drives performance in our task. Yet, an equally possible alternative interpretation is that it is expertise in combination with long-term vision loss that makes the difference. Either way, it is clear from the results that blindness alone does not lead to superior performance, but that even people who are blind need to have experience in echolocation in order to demonstrate behavioral benefits.

### Echolocation Acoustics, Expertise, and Kinematics Are Coupled During Adaptive Walking

Our report is the first to relate acoustics of clicks to movement kinematics, demonstrating how clicks may drive movement behavior. We found that people who walked faster also had a higher rate of clicking, higher peak frequencies, and briefer durations of clicks. Yet, splitting results into experts and beginners showed only the relationship between spectral content and walking speed applied to all participants, while only experts who walked faster also clicked at higher rates. The association between movement speed and clicking rate mirrors data from bats, where higher wingbeat frequency (and thus higher movement speed) is associated with higher emission rates (e.g., Holderied & von Helversen, 2003; Jones, 1994; Schnitzler, Kalko, Miller, & Surlykke, 1987). Bats also show marked changes in emission rates and peak frequencies, for example, when they approach a prey target (e.g., Ghose & Moss, 2006; Surlykke & Moss, 2000).

Using “‘static” perceptual tasks that did not require movement, we (Norman & Thaler, 2018; Thaler & Castillo-Serrano, 2016; Thaler et al., 2018) and others (Flanagin et al., 2017) have shown in previous research in human echolocation that acoustic features of clicks are related to performance. Crucially, the present study demonstrates the relation of acoustics to movement during walking.

**Figure 9 fig9:**
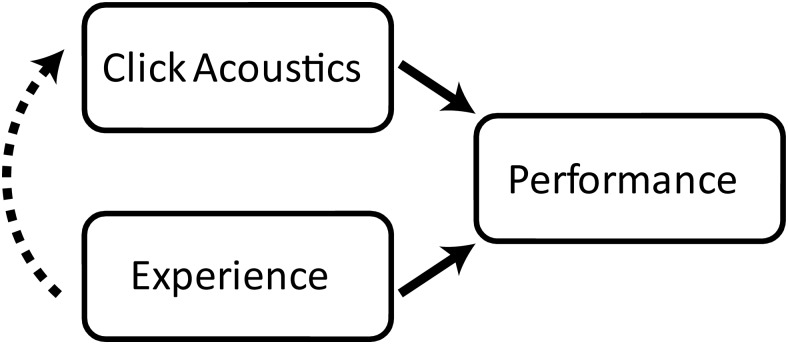
Illustration of our proposal of how click acoustics and click-based echolocation experience might jointly influence performance on click-based echolocation tasks.

Multiple regression analysis showed that experience in echolocation provides predictive power in addition to acoustic parameters. Figure 9 provides an illustration of how acoustics and experience may influence behavior in click-based echolocation tasks. The idea is that performance will depend both on the acoustic properties of clicks and experience, but that experience also influences click acoustics. Regarding the relationship between acoustic properties of clicks and performance, we suggest that, for example, clicks with higher spectral frequency content lead to better performance because higher spectral frequency content is associated with shorter wavelength, thus allowing better spatial resolution and leading to higher intensity echoes from targets of finite size (Norman & Thaler, 2018). Regarding the relationship between experience and performance we suggest that more experience leads to better performance because experience may influence the perceptual and/or cognitive aspects driving interpretation of echoes. For example, someone with more experience will have a better idea what a specific sound relates to in terms of the spatial environment because they have had more opportunity to establish links between sound and space. Regarding the relationship between experience and click acoustics, we suggest that, for example, people with more experience in echolocation will have developed clicks that have higher spectral frequency content, because they have had more opportunity to experience that brighter clicks will lead to stronger echoes, thus allowing them to “fine tune” their emissions. Furthermore, people with experience in echolocation show dynamic adaptation of their clicks (e.g., intensity, number of clicks) to compensate for weaker target reflectors (Thaler et al., 2018, 2019), and there is the possibility that situational adaptation of click acoustics in experts may be the result of having had more opportunity to discover that this is useful for performance.[Fig-anchor fig9]

The research and results presented here open an intriguing opportunity because they suggest that sensorimotor coupling for walking in people might not only be visually driven, but that echo-acoustics might serve the same function. There is a long tradition of investigating visual control for adaptive motor behaviors in humans (Fajen & Warren, 2003; Frenz & Lappe, 2005; Mohagheghi, Moraes, & Patla, 2004; Reynolds & Day, 2005). Echo-acoustic input that people obtain with click-based echolocation is temporally sparse, that is, people get intermittent “snapshots” of their environment. In comparison, input provided through vision is reasonably continuous. The question arises how two such very different forms of sensory input might serve similar functions. One possibility might be that either can serve as an updating mechanism for movement in space, for example, position of self with respect to the obstacle. There is other sensory information to drive such updates, for example, sensory information arising from vestibular and musculoskeletal sources (Chrastil, Sherrill, Aselcioglu, Hasselmo, & Stern, 2017; Day & Guerraz, 2007; Fitzpatrick, Wardman, & Taylor, 1999; Loomis, Klatzky, Golledge, & Philbeck, 1999). These can be used to update a person’s position within represented space. If such a process was in place, either vision or echo-acoustics would be an additional source of information feeding into these updating mechanisms. Within this process blind echo experts may have adapted nonvisual sensory sources to support echo-acoustic updates, while sighted people may have adapted the process to support visual updates.

Yet, it is important to emphasize that in our study echolocation did not act as a straightforward sensory substitution for vision. In our study, for example, the benefits of echolocation in terms of collisions were limited to head height obstacles, while people using vision had no collisions at all. Furthermore, while experts’ movement trajectories showed earlier obstacle avoidance and smoother movement paths than beginners, the trajectories between vision and echolocation were not equivalent. In this context it is important consider the nature of the sensory information arising from echolocation and vision. Echo-acoustic updates from mouth clicks arise intermittently, while in vision updates arise more continuously. Furthermore, the wavelength at which click-based echolocation operates is in the acoustic spectrum. Vision operates in the optic spectrum, with much shorter wavelengths which yield better spatial resolution. Along the same logic, we note that while vision provides approximately even coverage of the scene, echolocation works better for obstacles above the ground. The echo arising from a ground-level obstacle arises at about the same time as the echo of the ground itself. Furthermore both obstacle and ground were made from solid, smooth surfaces, and as such did not present much acoustic contrast. As such, detection of the obstacle against the ground will be significantly affected by backward masking. At head level, these effects are reduced, as there is a clearer contrast between obstacle and air and reduced masking. An interesting question in this context would be at which point echolocation might become a straightforward sensory substitution for vision, if at all. Based on the above, one prediction would be that performance should become increasingly equivalent as sensory samples across echolocation and vision become more similar. Consistent with this idea, we have found in the context of a perceptual distance judgment that the human brain will for example combine echolocation and vision in a statistically optimal fashion, that is, based on their reliability (Negen, Wen, Thaler, & Nardini, 2018).

In sum, the results presented here open the intriguing opportunity to investigate sensory motor coupling in people using echolocation, which has the advantage of easily quantifiable acquisition of sensory samples, thus permitting great transparency into the sensory sampling process.

### Practical Implications: Effects of Obstacle Elevation

In all participant groups, when people used echolocation only, collisions were more frequent with ground-level obstacles as compared to head-level obstacles. This is the first time that this effect has been experimentally demonstrated. While this demonstrates that echolocation increases safety by reducing collisions with obstacles at head height, one of the most common safety concerns for people with vision impairments (Williams, Hurst, & Kane, 2013), the data also clearly suggest that click-based echolocation was not effective for avoiding obstacles on the ground. In addition to the factors mentioned in the previous section, that is, masking which plays a larger role for obstacles placed on the ground as compared to obstacles suspended in air, the limiting aspect may also occur because of the way the click sound propagates from the mouth (Thaler et al., 2017), and because obstacles on the ground are further away from the mouth of a person walking or standing upright. It is for this reason that reflections from objects at lower positions will be fainter than reflections from obstacles at head height.

Our findings have important implications for instruction of potential echolocation users. Specifically, while it is worth emphasizing that click-based echolocation is useful for avoiding collisions with obstacles at head level, great care should be taken in creating awareness of comparably lower efficiency of echolocation for detecting obstacles on the ground.

### Practical Implications: Echolocation and the Long Cane—Synergy and Dual Tasking

Even without echolocation, it would be theoretically possible to locate obstacles at any level using acoustic echoes from ambient sound. Participants using only the long cane, however, were almost unable to locate the top obstacle in our experiment. This demonstrates that clicks were needed to detect head-level obstacles. Most importantly, in detecting obstacles at head height we found that in conditions where people used echolocation together with the long cane, collision frequency was the same as when echolocation was used alone. Vice versa, in detecting obstacles at ground level we found that in conditions where people used the cane together with echolocation, collision frequency was the same as when the cane was used alone. This implies that the combination of echolocation and cane did not impose any “costs” in terms of collisions. In other words, the use of echolocation in addition to the long cane (or vice versa) did not have any detrimental effect in comparison to either method being used alone with respect to collisions. In sum, our data suggest that the echolocation and long cane methods are complementary, and that the optimal strategy for navigation is to combine both methods.

In contrast, when we investigated movement speed we found that people who were new to echolocation not only walked more slowly than experts, but they walked even more slowly in conditions where they used echolocation either alone or in combination. This was not observed for experts. This suggests that in terms of movement speed echolocation may impose a “cost” on adaptive walking for people who are new to echolocation, but that this disappears with practice. Since this is the case both when echolocation is used alone, and in combination with the cane, it is not a cost of combining echolocation with the long cane (or vice versa), but it is a cost of using a new technique. In sum, people who are new to echolocation will slow down in their adaptive walking when using echolocation. This effect disappears in people who have expertise.

These findings have implications for mobility instruction as they suggest that higher probability of collisions for ground-level obstacles with echolocation can be compensated by simultaneous usage of the long cane, and vice versa, that lower probability of collisions for head- height obstacles with long cane can be compensated for by echolocation. At the same time our results clearly show that practice of echolocation is required in order to fully develop the skill, which, in our case, means that people who are new to echolocation will walk more slowly and slow down more when using echolocation either alone or in combination with the cane, but that this need to slow down will go away with experience in using this skill.

## Conclusion

We have provided data showing that the human action system can flexibly use click-based echolocation to support sensory-motor coordination in a task that is typically achieved using vision. Data from blind echo experts show that experience is needed to fully develop this skill, to the degree that the use of intermittent echo-acoustic samples may approach or parallel the use of visual information by sighted individuals to govern walking speed and paths. From an applied perspective, the data suggest that echolocation provides functional benefits to people who are blind, and also emphasize that echolocation should be combined with other mobility techniques (e.g., long cane) to increase detection of ground obstacles.

## Supplementary Material

10.1037/xhp0000697.supp

## Figures and Tables

**Table 1 tbl1:** Details of Blind Participants New to Mouth-Click-Based Echolocation

ID	Sex	Age (years)	Visual ability at testing	Age at onset of vision loss	Cause of vision loss
BC1	M	43	Totally blind	15	Bloodclot; Damage to optic nerve
BC2	M	51	Totally blind	birth	Retinitis pigmentosa
BC3	M	43	Totally blind	Birth	Ocular albinism
BC4	F	25	total right eye; left eye 1° of visual field	Birth	Rod-cone Dystrophy
BC5	F	46	residual central vision in right eye; total left eye	Birth	Unclear
BC6	M	36	bright light	Birth	Retinal dystrophy
BC7	M	68	bright light	Childhood	glaucoma
BC8	F	58	Some peripheral vision in right eye; total blindness left eye	birth; increasing severity	Stichler’s syndrome; Retinal sciasis
BC9	F	54	left eye total; right eye some periphery	Birth	Coloboma
BC10	M	76	Totally blind	birth; progressive; totally blind since 50 years of age	Retinitis pigmentosa
*Note.* BC = blind participants; M = male; F = female.					

**Table 2 tbl2:** Details of Blind Participants With Expertise in Mouth-Click-Based Echolocation

ID	Sex	Age (years)	Visual ability at testing	Age at onset of vision loss	Cause of vision loss	Use of mouth-click based echolocation
BE1	M	31	Totally blind	Gradual loss from birth	Glaucoma	Daily, since Age 12 years
BE2	M	49	Totally blind	Enucleated at Ages 7 and 13 months	Retinoblastoma	Daily, as long as can remember
BE3	M	33	Totally blind	Vision loss at Age 14 years	Optic nerve atrophy	Daily, since Age 15 years
BE4	F	39	Totally blind	Enucleated bilaterally at Age 22 months	Retinoblastoma	Daily, since Age 31 years
BE5	F	36	Total blindness in right eye; 1/60 vision in left eye	No sight at birth, given vision at Age 2 years	Microphthalmia, coloboma	Situational (low vision environments), since Age 29 years
BE6	M	51	visual field 4%	Vision loss started at Age 30 years	Retinitis pigmentosa	Situational (low vision environments), since Age 44 years
BE7	M	19	Totally blind	Vision loss at Age 36 months	Congenital amaurosis	Daily, as long as can remember
*Note.* BE = blind echolocation experts; M = male; F = female.						

**Table 3 tbl3:** Summary Statistics for Click Acoustics

Participant group	Rate (Hz)	Peak frequency (kHz)	Duration (ms)	RMS intensity (dB)
Echo-experts				
Mean (*SD*)	1.28 (.08)	2.56 (.22)	3.37 (.36)	−11.5 (1.22)
Min	.96	1.81	2	−14.63
Max	1.66	3.39	4.66	−6.98
Blind echo-beginners				
Mean (*SD*)	1.2 (.15)	1.92 (.15)	5.71 (.55)	−11.07 (1.04)
Min	.5	1.15	2.83	−16.1
Max	1.88	2.59	7.97	−5.25
Sighted echo-beginners				
Mean (*SD*)	1.01 (.11)	1.55 (.16)	17.33 (3.63)	−11.19 (.55)
Min	.38	.79	4.16	−15.02
Max	1.64	2.88	38.62	−8.15
*Note.* RMS = root mean square.				

## Footnotes

1 Please note that we also ran an analysis only considering trials without collisions. The pattern in the data across conditions/groups remains the same. The problem is that such an analysis gives us vastly unequal numbers of trials across conditions and also very low trial numbers in some conditions. Thus, from a statistical point of view, the analysis taking into account all trials is more appropriate and, considering that the pattern of results is the same, the current analysis is a valid description of performance in our study.
