# Supplementary material for: The Flexible Action System: Click-Based Echolocation May Replace Certain Visual Functionality for Adaptive Walking
Source: J Exp Psychol Hum Percept Perform. 2019 Sep 26;46(1):21–35. doi: 10.1037/xhp0000697 (PMC6936248; doi:10.1037/xhp0000697)
Supplement: Supplementary file 1 [file Thaler_et_al_SEM_xhp0000697.docx]

The Flexible Action System: Click-Based Echolocation May Replace Certain Visual Functionality for Adaptive Walking

Lore Thaler^1^, Xinyu Zhang^2^, Michail Antoniou^3^, Daniel C. Kish^4^, Dorothy Cowie^1^

1 - Department of Psychology, Durham University, UK

2 - School of Information and Electronics, Beijing Institute of Technology, China

3 - Department of Electronic Electrical and Systems Engineering, University of Birmingham, UK

4 - World Access for the Blind, Placentia, California, United States

Corresponding author:

Lore Thaler

[lore.thaler@durham.ac.uk](mailto:lore.thaler@durham.ac.uk)

Department of Psychology, Durham University

Science Site, South Road

Durham DH1 3LE

United Kingdom

SUPPLEMENTAL MATERIAL

Below we provide a detailed description of the training procedure employed for participants taking part in echolocation conditions.

First, the experimenter demonstrated a mouth click made by pressing the tongue against the roof of the mouth (palate) and then quickly pulling away a part of the tongue to create a vacuum pulse. Whilst making this tongue motion the experimenter also made a wide open smile to facilitate the clicking process by stabilizing cheek and jaw muscles and permitting the sound to come out of the mouth. Participants were then asked to copy the sound using their tongue and mouth. Some of them just did this naturally. But some expressed uncertainty as what to do. In this case the experimenter verbally described the movements that the different parts of the tongue and mouth should be making (i.e. tongue against roof of mouth and pulling away, wide open smile). If a participant wanted further clarification, the experimenter also used their own hands as well as the hands of the participant to model the motions using touch. They also demonstrated the effect the shape of the mouth opening on emitted sound, i.e. they made clicks forming an ‘o’ with their lips or an open wide smile (like for saying ‘e’). Once participants said that they had an idea what was asked of them they were then instructed to make their own clicks. They then practiced clicking for ~5 minutes. Once they were happy with the clicking process, they were then led to the centre of the room. At the centre of the room the experimenter held the object used as an obstacle in the main experiment at the participants head height and facing them at a distance of ~5 cm. The participant was asked to lift one of their hands so that they could feel the bottom of the object when it was placed in front of them. Then, they were asked to make clicks. Whilst making clicks, the object was lifted up, so that the space in front of the participant was clear, and participants could feel with their hand that the object had been lifted because the bottom of the obstacle was not touching their hand anymore. The experimenter then asked the participants if they could hear the difference between the object being in front of them or not. This was repeated until the participant reported to be confident hearing the change in sound when the object was removed. At this point, the experimenters explained that the participant should now lower their hand, but continue to make clicks, and that the experimenter would now start out by holding the object in front of them, and then move it away. The participant’s task was to lift their hand if they felt that the object had been removed. Once the participant had successfully indicated this change three times in a row, the same task was repeated, but the experimenter started out with the object removed, and then brought it in front of the participant. The task of the participant was now to raise their hand when they felt that the object appeared in front of them. Once they had made this judgment correctly three times in a row the experimenter suspended the obstacle from the ceiling at the participants head height. They then asked the participants to touch the object to get tactile feedback about its location. Then, participants were asked to make clicks and to take side steps and to stop when they had the feeling that the object was no longer in front of them, and then to reach out to get feedback about the object’s location. They were asked to repeat this process until they did it correctly three times in a row. After this, they were led ~2m away from the obstacle, facing the obstacle. They were then asked to walk ahead towards the obstacle and to stop when they felt that something was in front of them. If they did not stop early enough they received feedback from the touch of the object. If they stopped early enough they were asked to reach out to get feedback about the object location. Participants were told that if they were not sure if there was something in front of them or not, they could rotate their head to the side and click to the side of the room. Since there would never be an object placed to the side, they could then use that as a ‘reference’ against which to compare the sound from the front. Once participants had managed to stop in front of the obstacle twice without touching it, they were then asked to do the same, but without stopping and reaching. Instead, they were instructed to walk past the object without touching it with any part of their body. Once they had done this successfully two times the echolocation practice was concluded.
